# Correction: Wang et al. Integration of Transcriptomics and Lipidomics Profiling to Reveal the Therapeutic Mechanism Underlying *Ramulus mori* (Sangzhi) Alkaloids for the Treatment of Liver Lipid Metabolic Disturbance in High-Fat-Diet/Streptozotocin-Induced Diabetic Mice. *Nutrients* 2023, *15*, 3914

**DOI:** 10.3390/nu16244285

**Published:** 2024-12-12

**Authors:** Fan Wang, Sai-Jun Xu, Fan Ye, Bin Zhang, Xiao-Bo Sun

**Affiliations:** 1 Institute of Medicinal Plant Development, Peking Union Medical College, Chinese Academy of Medical Sciences, Beijing 100193, China; wangfan@implad.ac.cn (F.W.); xsj365fighting@163.com (S.-J.X.); spring2378@163.com (F.Y.); 2 Key Laboratory of Bioactive Substances and Resources Utilization of Chinese Herbal Medicine, Ministry of Education, Beijing 100193, China; 3 Beijing Key Laboratory of Innovative Drug Discovery of Traditional Chinese Medicine (Natural Medicine) and Translational Medicine, Beijing 100193, China; 4 Key Laboratory of Efficacy Evaluation of Chinese Medicine Against Glyeolipid Metabolism Disorder Disease, State Administration of Traditional Chinese Medicine, Beijing 100193, China

In the original publication [1], there was a mistake in the published version of Figure 3B (SZ-A 50 mg/kg group). In Figure 3B, an error occurred during the assembly, with the incorrect SZ-A 50 mg/kg image from our correction, which is now replaced with the correct image. The corrected version of Figure 3 appears below.

The authors apologize for any inconvenience caused and state that the scientific conclusions are unaffected. This correction was approved by the Academic Editor, and the original publication has also been updated.

## Figures and Tables

**Figure 3:**
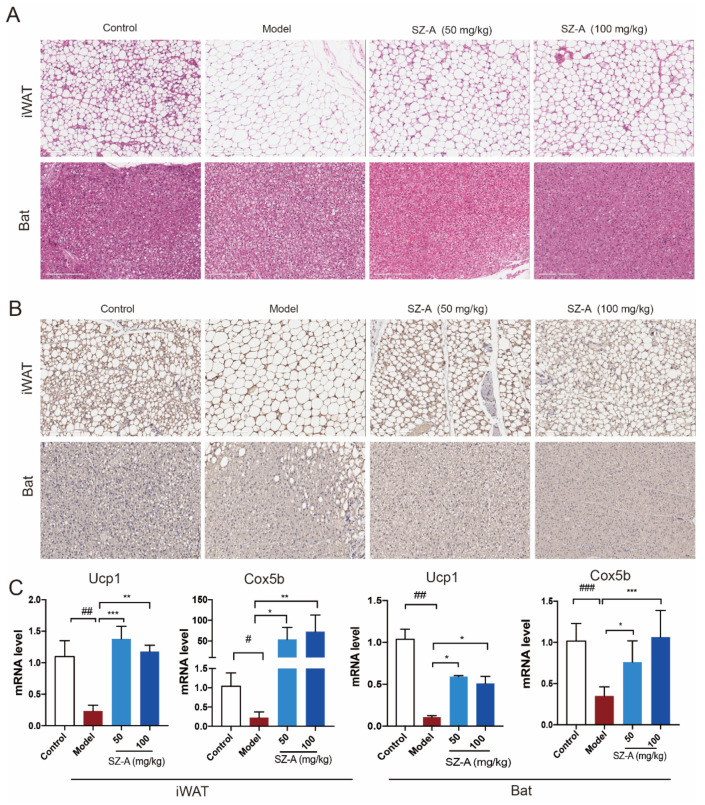
Adipose metabolic profiles and thermogenesis levels of HFD/STZ-induced mice after SZ-A treatment. Representative hematoxylin and eosin (H&E) staining images (**A**) and Ucp1 immunohistochemical staining images (**B**) of mouse iWAT and Bat, scale bar: 200 μm. (**C**) RT-qPCR analysis of Ucp1 and Cox5b expression in iWAT and Bat (*n* = 6/group). The data represent the means ± SD. # *p* < 0.05, ## *p* < 0.01, ### *p* < 0.001 versus the control; * *p* < 0.05, ** *p* < 0.01, *** *p* < 0.001 versus the HFD + STZ.
